# Supplementary material for: Early life exploration behaviour and life‐history loci are colocalized in an adaptive genomic hotspot in Atlantic salmon
Source: J Anim Ecol. 2025 Dec 5;95(3):418–29. doi: 10.1111/1365-2656.70197 (PMC12957736; doi:10.1111/1365-2656.70197)
Supplement: Supplementary file 1 — Table S1. Age and hatch year of individuals used in this study across populations (Teno, Inarijoki) and their main stems and small tributary (nursery) streams. The numbers in parenthesis are smolts. Table S2. Observed number of individuals in the Inarijoki population, main stem (0) vs. the Guoldnájohka stream (1) in relation to akap11 and vgll3 TOP genotypes. Figure S1. The study system and the Teno River Basin. River sections with blue and red colours indicate Teno main stem and Inarijoki, respectively, and respective nursery streams, Baððá and Guoldnájohka. Note that the sampling in both spawning rivers were carried out in close proximity (up to 100 m) to the outlets of two nursery streams. Figure S2. Genotyping success per individuals (a) and SNPs (b). Focal SNPs are marked in red in (a). Excluded individuals were marked in red in (b). Figure S3. Parsimony of different model structures as evaluated by Akaike information criteria (AIC). Models in the grey shaded area indicates similarly parsimonious model (i.e. models within 2 AIC units to the best model). Figure S4. Predicted odds of exploration in Inarijoki Atlantic salmon as a function of akap11 and vgll3 interaction with all genotype combinations are visualized. Error bars indicate 95% CI of the marginal estimates and numbers in parenthesis indicates sample size. Y axis is drawn in log scale. Figure S5. Diagnostic plots of the models that has only akap11 as the genotype factor (a) and that modelled akap11 and vgll3 interactions. Figure S6. Comparing the parsimony of model structures between modelled with and without smolts included in the dataset (dark vs. light symbols, respectively), as evaluated by Akaike information criteria (AIC). Models in the grey shaded area indicates similarly parsimonious model (i.e. models within 2 AIC units to the best model). Figure S7. 1000 permutated models to evaluate the effect of including or exluding smolts (individuals that had undergone parr‐smolt transformation) to the pa [file JANE-95-418-s001.docx]

Supplementary Tables (2) and Figures (7) for

Early life exploration behavior and life-history loci are co-localized in an adaptive genomic hotspot in Atlantic salmon.

**Supplementary Table 1:** Age and hatch year of individuals used in this study across populations (Teno, Inarijoki) and their main stems and small tributary (nursery) streams. The numbers in parenthesis are smolts.

|  |  |  | Hatch year | | | | | |
| --- | --- | --- | --- | --- | --- | --- | --- | --- |
|  |  | Age | 1988 | 1989 | 1990 | 1991 | 1992 | 1993 |
| Teno | main stem | 1 | 0 | 0 | 10 | 0 | 0 | 0 |
|  |  | 2 | 0 | 36 | 0 | 0 | 0 | 0 |
|  |  | 3 | 21 | 0 | 0 | 0 | 0 | 0 |
|  |  |  |  |  |  |  |  |  |
|  | Baððá | 1 | 0 | 0 | 0 | 0 | 5 | 50 |
|  |  | 2 | 0 | 0 | 0 | 6 | 52 | 0 |
|  |  | 3 | 0 | 0 | 5 | 18 (4) | 0 | 0 |
|  |  | 4 | 2 (2) | 9 (5) | 44 (43) | 0 | 0 | 0 |
|  |  | 5 | 18 (17) | 14 (14) | 0 | 0 | 0 | 0 |
|  |  |  |  |  |  |  |  |  |
| Inarijoki | main stem | 1 | 0 | 0 | 0 | 0 | 14 | 5 |
|  |  | 2 | 0 | 14 | 0 | 21 | 15 | 0 |
|  |  | 3 | 16 | 0 | 5 | 6 | 0 | 0 |
|  |  |  |  |  |  |  |  |  |
|  | Guoldnájohka | 1 | 0 | 0 | 15 | 4 | 3 | 22 |
|  |  | 2 | 0 | 28 | 0 | 12 (1) | 34 | 0 |
|  |  | 3 | 4 | 0 | 8 | 7 | 0 | 0 |
|  |  | 4 | 0 | 61 (28) | 51 (48) | 0 | 0 | 0 |

**Supplementary Table 2**: Observed number of individuals in the Inarijoki population, main stem (0) vs the Guoldnájohka stream (1) in relation to *akap11* and *vgll3*_TOP_ genotypes.

| *akap11* | *vgll3*_TOP_ | 0 | 1 |
| --- | --- | --- | --- |
| EE | EE | 22 | 87 |
| EE | EL | 32 | 92 |
| EE | LL | 3 | 24 |
| EL | EE | 4 | 0 |
| EL | EL | 26 | 30 |
| EL | LL | 3 | 13 |
| LL | EE | 0 | 0 |
| LL | EL | 4 | 1 |
| LL | LL | 2 | 2 |

**
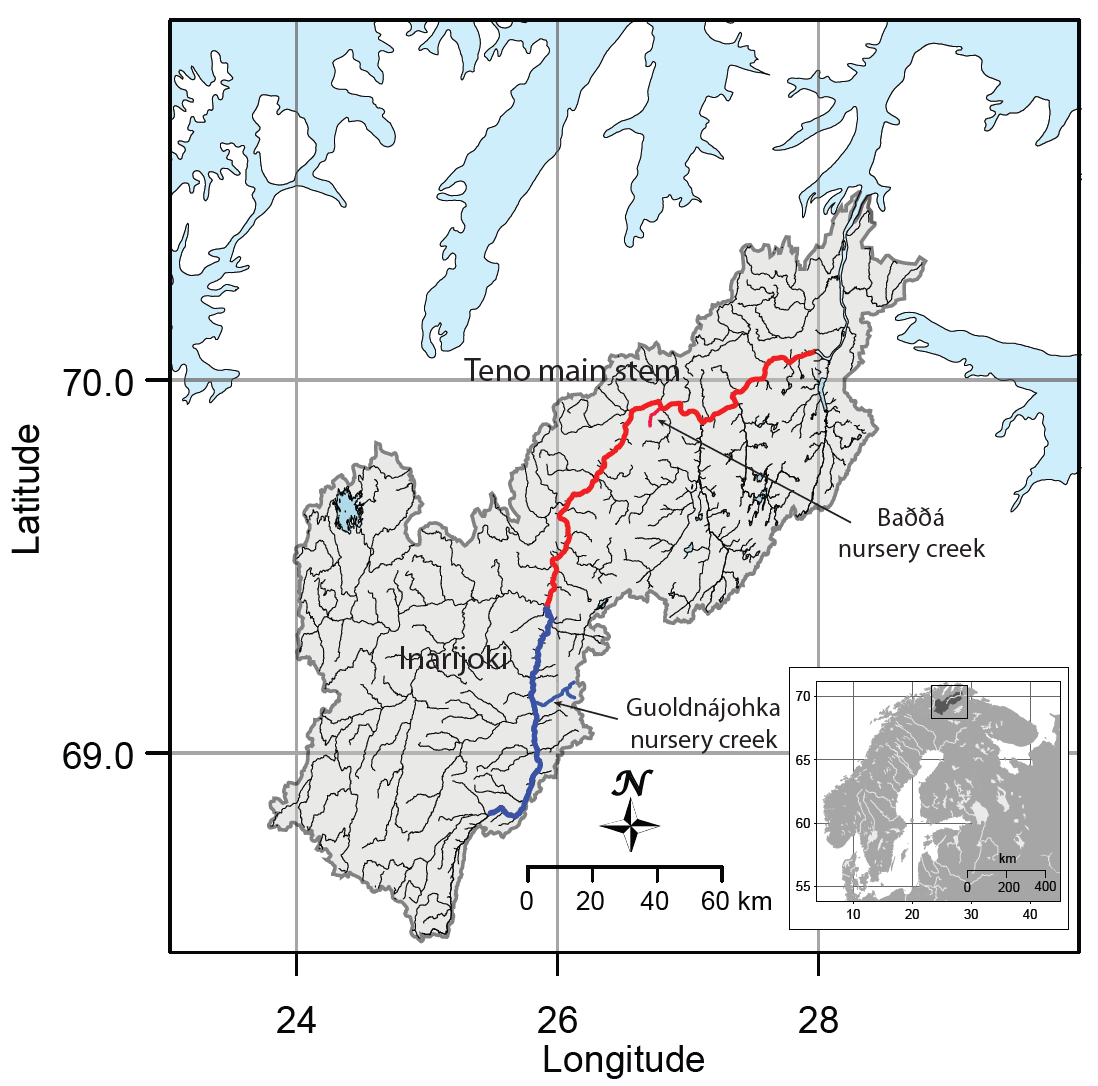
**

**Supplementary Figure 1:** The study system and the Teno River Basin. River sections with blue and red

colors indicate Teno main stem and Inarijoki, respectively, and respective nursery streams, Baððá and Guoldnájohka. Note that the sampling in both spawning rivers were carried out in close proximity (up to 100 meters) to the outlets of two nursery streams.


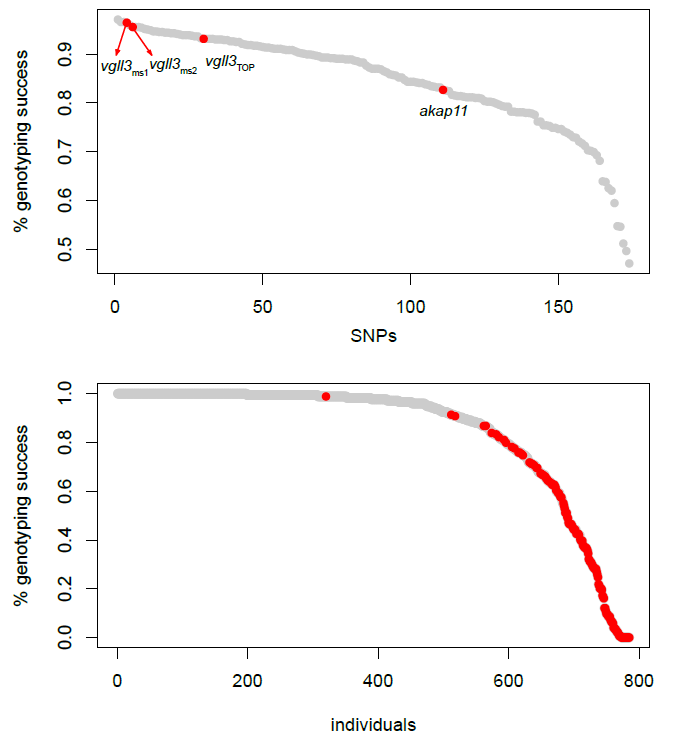


**Supplementary Figure 2:** Genotyping success per individuals (a) and SNPs (b). Focal SNPs are marked in red in (a). Excluded individuals were marked in red in (b).


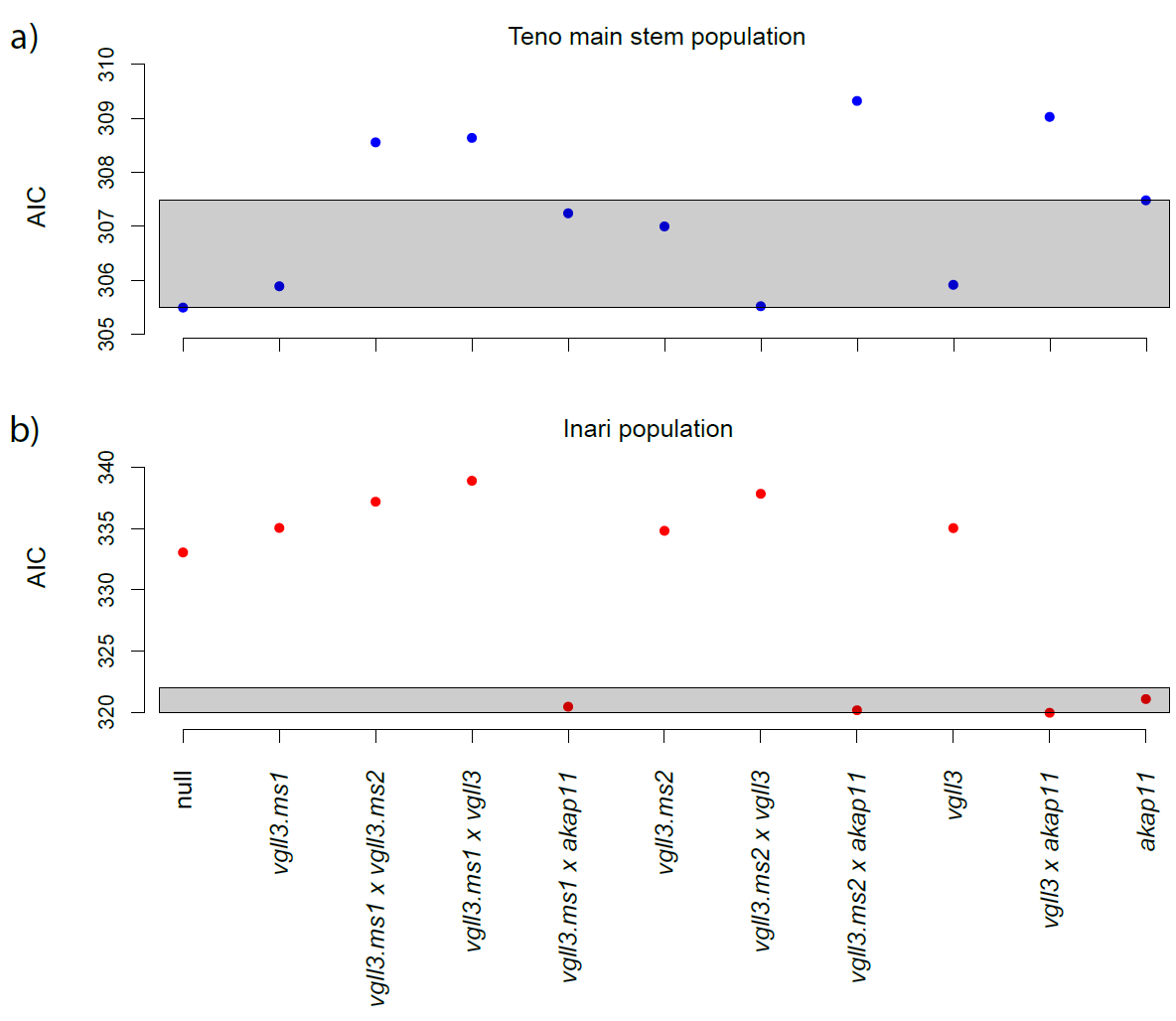


**Supplementary Figure 3:** Parsimony of different model structures as evaluated by Akaike information criteria (AIC). Models in the gray shaded area indicates similarly parsimonious model (i.e., models within 2 AIC units to the best model.)


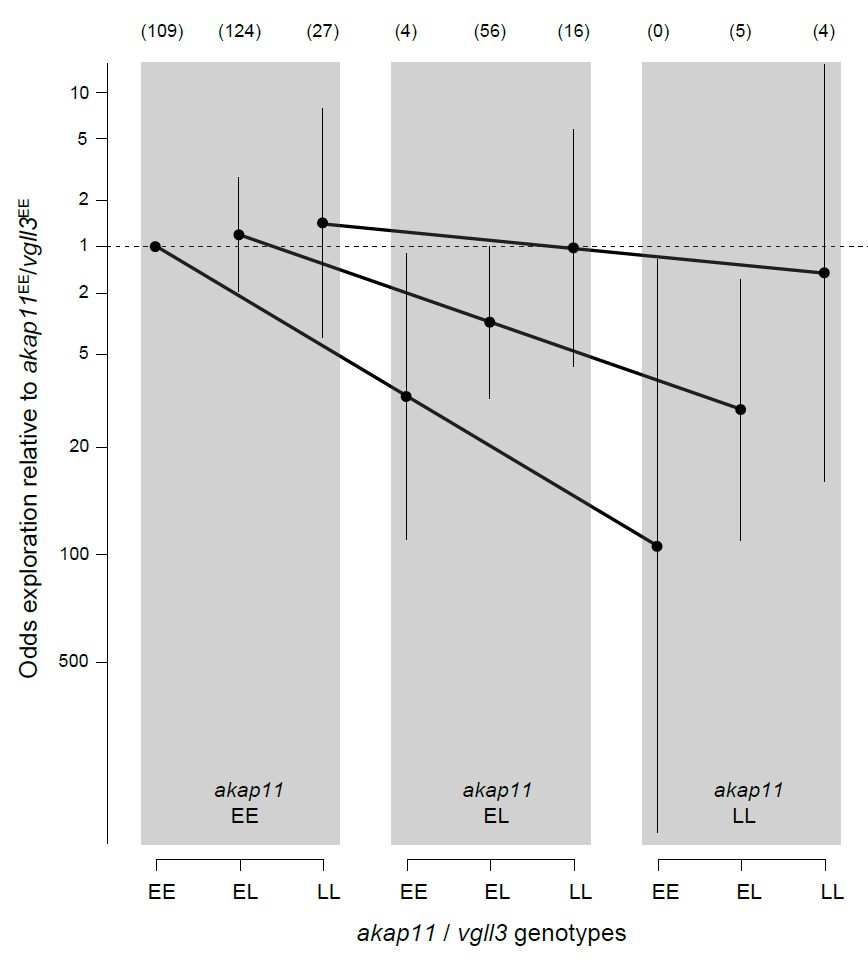


**Supplementary Figure 4:** Predicted odds of exploration in Inarijoki Atlantic salmon as a function of *akap11* and *vgll3* interaction with all genotype combinations are visualized. Error bars indicate 95% CI of the marginal estimates and numbers in parenthesis indicates sample size. Y axis is drawn in log scale.


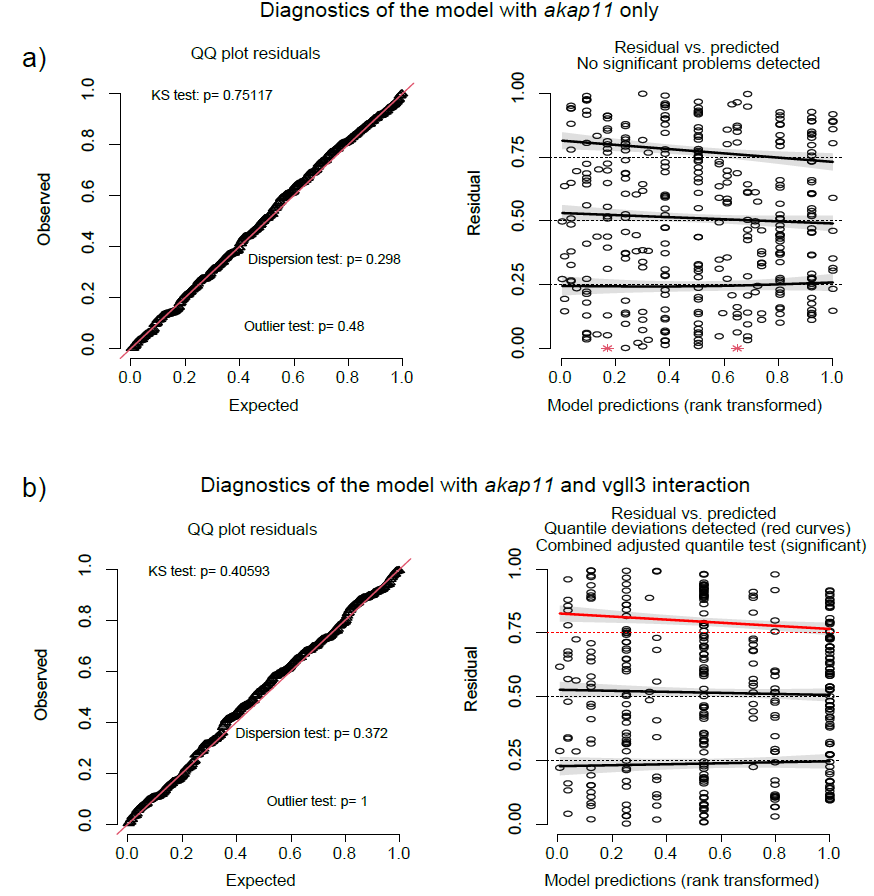


**Supplementary Figure 5:** Diagnostic plots of the models that has only *akap11* as the genotype factor (a), and that modelled *akap11* and *vgll3* interactions.


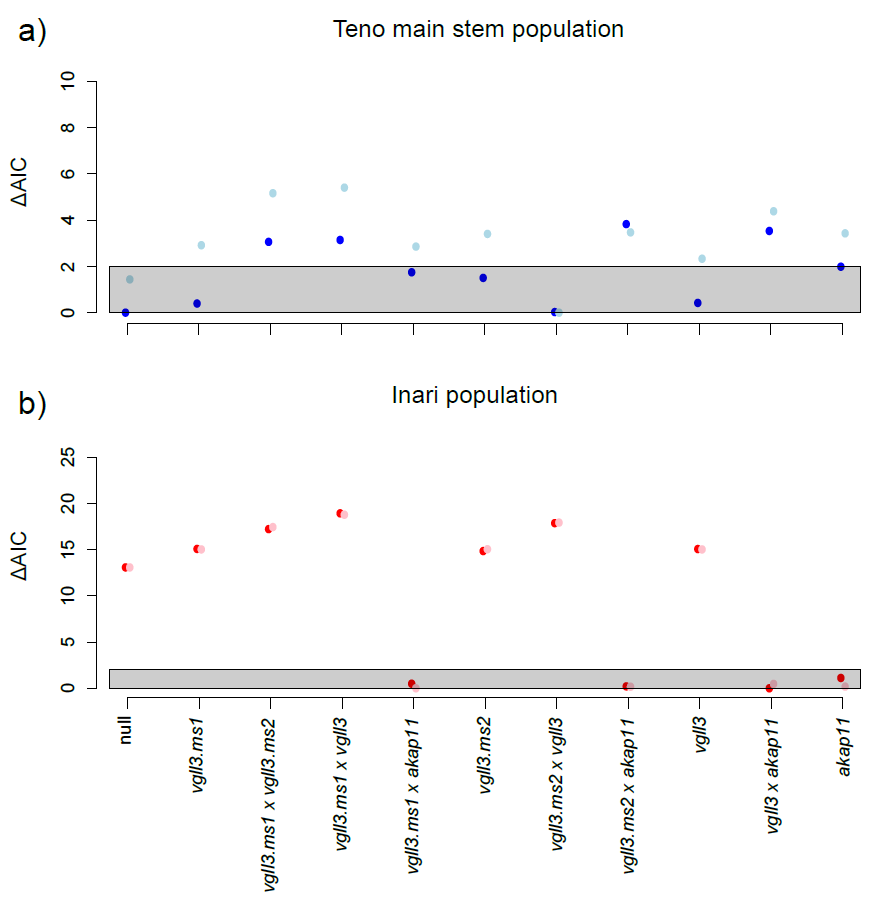


**Supplementary Figure 6:** Comparing the parsimony of model structures between modelled with and without smolts included in the dataset (dark *vs.* light symbols, respectively), as evaluated by Akaike information criteria (AIC). Models in the gray shaded area indicates similarly parsimonious model (i.e., models within 2 AIC units to the best model.)


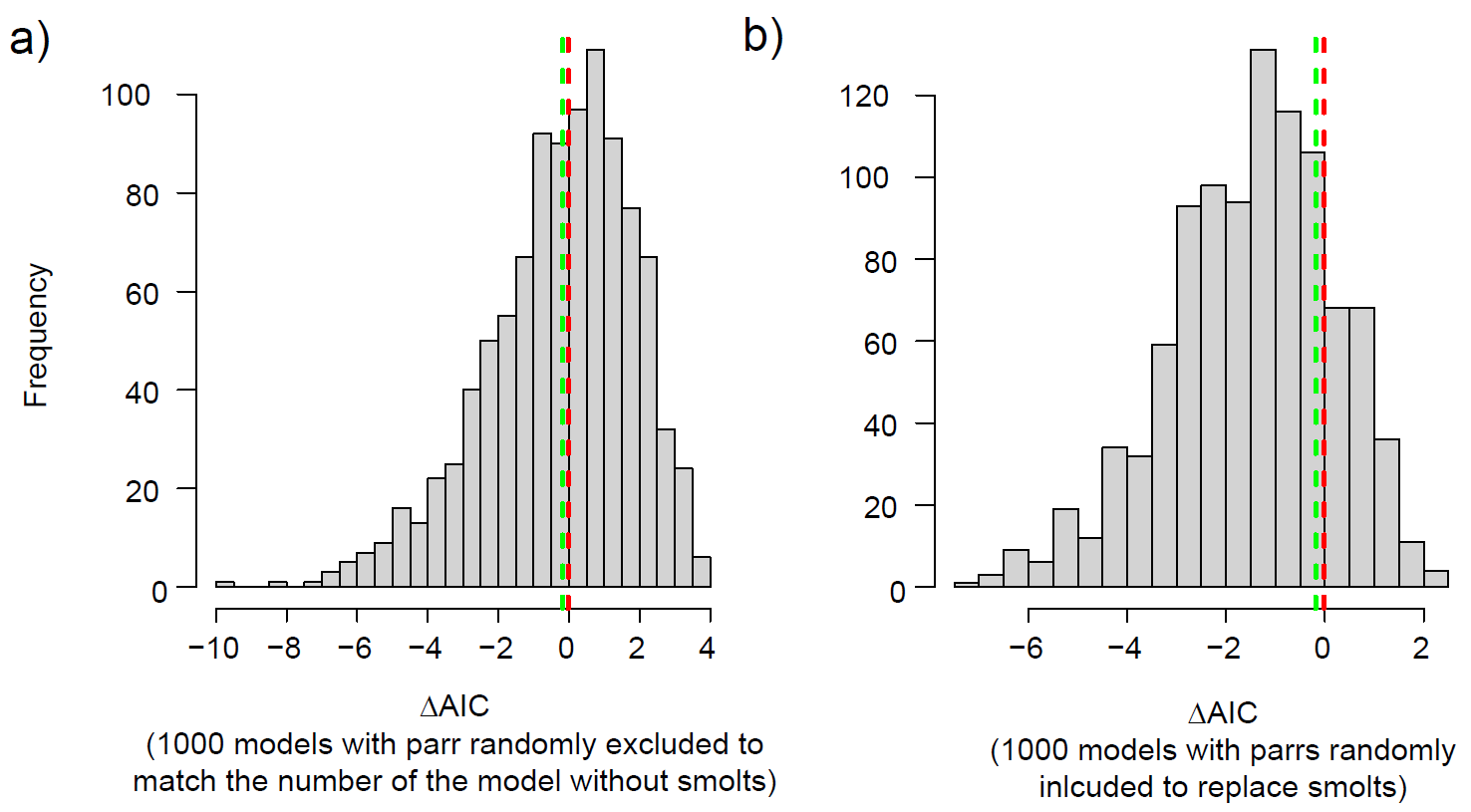


**Supplementary Figure 7:** 1000 permutated models to evaluate the effect of including or excluding smolts (individuals that had undergone parr-smolt transformation) to the parsimony of (ΔAIC) *akap11* additive vs. *vgll3*_TOP_ x *akap11* interaction models. In (a), equal number of parrs as the number of smolts were randomly excluded from the dataset, and parsimony between two model structures were compared, i.e., between the real model that excludes smolts (red dashed lines) and 1000 permuted models that randomly excluded parr. In (b), equal number of parrs as the number of smolts in the data were included to the dataset in exchange for excluding smolts, and parsimony between two model structures were compared, i.e., between the real model that still include smolts (red dashed lines) and 1000 permuted models.
